# A Collaborative Response to Provocative Comments in a Medical School

**DOI:** 10.15694/mep.2019.000199.1

**Published:** 2019-11-06

**Authors:** Kimberly R Myers, Rahima Khatun, Ghazal Staity, Shreela Natarajan, Daniel R George, George F Blackall

**Affiliations:** 1 Penn State College of Medicine

**Keywords:** provocative speech, student-faculty collaboration, Islamophobia, refugee, cybersecurity

## Abstract

This article was migrated. The article was marked as recommended.

In 2018, anonymous online provocative comments were submitted to student leaders of a Syrian Refugee Initiative (SRI) at the Penn State College of Medicine. This triggered a series of actions with students and medical school leaders aimed at identifying the person who submitted the comments, trying to understand mutual perspectives, and managing the impact of the event on the student body.

We describe the history of our college’s commitment to humanism and how the SRI was a direct outgrowth of that culture. Voices of the student leaders who were directly impacted by the provocative comments and educational leaders who worked to resolve the crisis are presented. We also describe a collaborative process that involved engaging cybersecurity experts to identify the perpetrator, and share how the students and educational leaders were able to develop trust despite initial skepticism by students over the leadership’s avowed commitment to taking the hate speech seriously.

While the perpetrator was never identified and opportunities for improvement were identified along the way, by including student leaders in the process, students and administrators were able to develop trust and reach reasonable closure on this disturbing event. Take home messages are presented to guide other institutions in navigating instances of provocative comments or speech.

## The Inciting Incident

### Rahima, MS2, Co-leader of Syrian Refugee Initiative

It was Sunday, September 16 ^th^ at noon-the day after I had posted the volunteer application on the “Penn State College of Medicine Class of 2022” Facebook page for the Syrian Refugee Initiative (SRI) group I co-lead. I was reaching out to our new class of medical students, in hope of finding more people to support the families we work with. When I checked the volunteer application Google form to see if we had any new signees, it caught my attention:

**Name:** “no more terrorist scum”

**Email:** “average@american.com”

**Please provide a brief description of why you are interested in being involved as a 1 ^st^-year representative for the Refugee Initiative:** Enough of bringing these terrorist scum to the USA.

I read this over and over again. My first thought was “Is this a joke?” I immediately took screenshots of the submission and sent it to the other members of the board, partly to ask whether this was meant to be funny and partly to share my anger as I felt myself shaking with rage. Whoever this was and whatever the intention might be, this person was potentially from our community and had ill-informed and malignant views toward a group of people s/he had probably never met.

I had been working with eleven Syrian families for the past year and they had touched my heart. Every one of them had a story to share about fleeing their homes and building temporary homes in refugee camps-in Turkey, Jordan or Greece-in a land that was not theirs. They had come here, to the “land of opportunity”, as America is known to immigrants, with hopes of starting a new life. They had accepted hardship as their destiny and traveled across the world, only to be met by people who branded them “terrorists” based solely on their faith/ethnicity.

### Shreela, MS2, member of Syrian Refugee Initiative

I remember seeing the post on the group chat for our executive board. Initially I laughed because I thought it completely ridiculous, almost surreal. But as I kept reading, I began getting angry. This kind of anger had a sense of responsibility to it and made me want to go out and do something.

I have heard inappropriate comments made to students of our Muslim community, and I have witnessed the reaction of people on the receiving end of those comments. I am and have always been aware of the fact that ignorance and hate will exist in any community no matter where you are in the world. But to have something like this said in our *medical* community, *my* medical school, my place of *education*, felt like it wasn’t just one individual who was misguided; it felt like the whole community had failed.

### Ghazal, MS2, Co-leader of Syrian Refugee Initiative

Ironically, I wasn’t shocked when I first read the message. As a Syrian, I had personally encountered many situations like this in the past, and I understand that many people don’t share the goal of serving others, as my friends and I do, through practicing medicine.My lack of surprise is not to say, however, that uttering such horrific words about anyone can be condoned.Only a person who is ignorant of what a war can do to a country and its citizens would say such things about refugees who came here because there was no other option.They came here to escape death.Refugees are human, just like the rest of us.I had come to understand just how “human” they are as I visited their homes, ate their food, played with their kids, and witnessed how they were treated.It was not uncommon to hear stories of girls whose scarves had been pulled from their faces and heads.

### Rahima

I just sat there staring at the screen and recalled how this was the second incident of Islamophobia I had experienced in medical school. I had believed that since we were all pursuing a career in medicine, we would be open-minded and respectful toward others who appeared to be different. Obviously this was not the case. Earlier in the year, a classmate shared a first impression of me, stating that he had felt threatened by my hijab and thought I was a radical. For a while, I felt a sense of isolation and lack of belonging in this community. Were people just ignorant because of lack of exposure to other head-covered Muslims? Or was this a manifestation of the hatred and racism rampant in our country today, allowing others to lash out from the shadows with no repercussions for their slurs? Thinking about Islamophobia and how others perceived me as a woman wearing a hijab was not what I had envisioned would be my biggest struggle in medical school. I feared that others, too, saw me as a radical, stereotyping me based on my appearance. This incident felt like a direct attack on me and my faith. It was very disturbing to be accused of indirectly supporting terrorism.

### Ghazal

I actually feel sorry for this person who thought s/he could challenge our cause behind anonymous online templates, and I feel even sorrier that s/he believed s/he is entitled to make such a statement. Such action shows ignorance, cowardice, and a basic lack of human compassion. I would’ve gladly talked to anyone about these families that we help. In fact, we often share their stories at fundraising dinners and lunch lectures.

### Rahima

It was maddening to feel that I had no one I could direct my anger to or even confront and ask why s/he felt this way toward refugees. I was frustrated at the system: why aren’t there platforms for discussing, combatting, and dealing with things like prejudice and bigotry? Penn State emphasizes valuing diversity and fostering humanism in medicine, so I was dismayed to think such a person could be accepted into our medical school, much less care for future patients. I knew that I had to bring this problem to the attention of our leaders. I wanted to see exactly how important diversity, tolerance, and a respectful learning environment was to our deans, so I sent a mass email to all of them, hoping they would act.

## Context

### Daniel R. George, PhD, Faculty Advisor of Syrian Refugee Initiative

The Penn State College of Medicine (PSCOM) is located in Hershey, PA, a community of 14,000 people, 15 miles east of the state capital, Harrisburg. 580 medical students, 200 graduate students, 60 physician assistant students and roughly 1,000 faculty members comprise the PSCOM community; a 548-bed hospital with a level I trauma center is connected to PSCOM.

With a strong emphasis on the humanities and patient-centered care, PSCOM has a commitment to serving the surrounding community. Part of that mission is embodied in the Syrian Refugee Initiative (SRI), a medical student-led outreach project that was launched three years ago in response to the refugee crisis caused by ongoing war in Syria. In November 2016, eleven refugee families were resettled in the Harrisburg area. Given the food security challenges commonly faced by refugees, a group of students and their faculty mentors in a “Food as Medicine” group partnered with the state’s Refugee Health Program to involve families in weekly visits to a farmers’ market centrally located in Harrisburg. Using seed-grant funding, students carpooled mothers and children from their new homes to the market each Friday where the students helped purchase enough fruits and vegetables to last the week. Although the main intention was to ensure that families were meeting basic nutritional needs, students also felt a farmers’ market was a safe, welcoming venue to begin introducing people to their adoptive community ( Bouhman, Boothe, and George, 2017).

Students gradually expanded their mission, forming collaborative partnerships with Catholic, Jewish, and Islamic organizations, staff and students at our hospital, and other community groups to collect donations that met other needs (e.g., clothes, money, appliances). Students provided transportation to medical appointments and errands, offered translational help (e.g., medical paperwork, English training), developed a tutoring initiative to help children learn English, and organized several cultural exchange events to help raise funds for the families. The bonds with the Syrian families remain strong and lasting. This initiative reflects the kind of inclusive, compassionate behavior of most medical students. Such benevolent outreach is a natural extension of a profession whose mandate-and reward-is to serve individuals in need, regardless of race, custom, or creed.

## The Process

### George F Blackall, Psy.D., Director of Office for a Respectful Learning Environment

The Office for a Respectful Learning Environment (ORLE) at PSCOM was formed in 2016, in response to national awareness of and concern about medical student mistreatment. The ORLE Director is a psychologist whose responsibilities include responding to complex issues that arise in the learning environment. The provocative comments directed at the refugees triggered immediate attention.

Upon being contacted by the student leaders of SRI about the comments, my reaction was disgust and concern-disgust that any person could hide behind anonymity on a computer screen and write such abhorrent statements, concern for the SRI students to whom this was directed and, indeed, the entire student body.

I wanted to convey support by taking swift and decisive action and by building a collaborative connection with the SRI leaders.I invited The Penn State Health and College of Medicine Office of Cybersecurity to participate in a meeting with SRI leaders so that we could discuss whether Cybersecurity may be able to provide assistance in identifying the person who posted the comments. We recognized this was going to be a complex and potentially unsuccessful task, however Cybersecurity became engaged in this matter, and conducted an analysis.

At this meeting, student leaders expressed doubt about whether educational leaders were taking the events seriously. While there was a whirlwind of activity among our leadership team when the event occurred, we made a decision not to publicize the event or the work being done behind the scenes, in order to avoid compromising the investigation. If the perpetrator were a student, for instance, we did not want that person to know we were making inquiries and thereby potentially allow the person to cover his or her tracks. In addition, we did not want to trigger widespread suspicion about who might have submitted the comments, particularly since they could potentially have come from someone outside PSCOM. Students interpreted this silence as apathy on the part of leadership. When I shared these reasons with the students, they were reassured that this incident was, in fact, a priority.

Upon completing its investigation, Cybersecurity found no indicators that Penn State Health or College of Medicine computing resources were used to provide the comments that were made. In a last-ditch effort to identify the person who submitted the comments, a faculty member (KM) who had a personal connection to a project manager at Google, reached out to ask for help. The response was simply that without a search warrant, Google was unwilling to help.

Although reassured that Penn State resources had not been utilized, in light of fact that we were no closer to determining who posted the comments, SRI leaders felt it would be helpful for the Vice Dean for Educational Affairs; the Associate Dean for Diversity, Equity and Inclusion; and me to lead meetings with the first- and second-year classes. The purpose of the meetings would be to explain what had happened, convey that the event was taken seriously, describe the investigation, and communicate that this type of behavior is unacceptable.

## Class Meetings

We recognize that class meetings can carry risks. History at our institution has taught us that in large-group settings, a well-intended message can be interpreted in many unintended ways. Our biggest concern was that our efforts would be seen as inadequate or that we would incite a “mob mentality” wherein students would become suspicious of one another.

The initial class meeting was with the second-year class. At this time the full investigation was not complete, so there was hope-though limited-that the perpetrator would be identified. I began the meeting by presenting the actual comments and explained the investigative process and its findings. With support from the Associate Dean for Diversity, Equity and Inclusion, the Vice Dean for Educational Affairs then spoke about the impact of such events on our learning environment.

All went well until one student asked what would happen to the perpetrator if s/he were identified as a PSCOM student. In retrospect, this was an obvious question, but at the time, we had not anticipated it and therefore did not have a clear answer. The essence of the response was that, as a learning institution, we would have to take an approach based first on understanding-in particular trying to first comprehend what motivated the person to write the comments. Then, getting the person professional help and requiring him/her to develop a performance-improvement plan. This response angered students who felt it was too “soft.” Students asked pointedly whether the person could remain in our learning environment. The response was that the Academic Progress Committee, which oversees accountability for student competency in aspects of academic performance, would have to make a determination.

From a perspective that students might not immediately consider, educational leaders understand that the issue of “What’s the consequence?” is complicated because every situation has unique factors that influence consequences. Recognizing that such variables exist, PSCOM utilizes a specific process through the Academic Progress Committee to ensure accountability for all school-wide competencies, including professionalism, and address deficiencies in student behavior (e.g., students failing to complete academic or clinical responsibilities). One of the frustrating elements in this case was that the author of the unprofessional comments could not be identified.

We recognized that the feeling in the room at the end of the meeting was unfavorable. I met with SRI leaders the following day to get their feedback. Leaders were frank about their disappointment and frustration over lack of a clear message about consequences if the perpetrator were identified as a fellow student. The SRI leaders’ candor was helpful to us as it informed preparation for the meeting with the first-year class the following day.

The meeting with the first-year class was different in tone. We were clear in stating that if the person were identified as a COM student, a specific process would be implemented to understand why the student posted such bigoted views and to help him or her grow from the experience.If those efforts were not successful, the person would be referred to the Academic Progress Committee to determine whether the student had exhibited deficiencies in those program competencies within the purview of the APC, which may result in recommendations for remediation activities or other actions. Students at the meeting were vocal about their rejection of such hatred and expressed support for SRI leaders and appreciation for the leadership’s efforts to resolve the case.

### Shreela

In the end, after much reflection, I realized that it wasn’t the number of responses or the words that mattered. It was how genuine the response was. To have worked with a group of passionate faculty members has shown me that there is a way to make things right, one step at a time, one meeting at a time, and one discussion at a time. What we need is a conversation.

Slowly, my anger has fallen away and my skepticism has faded. What is left is a sense of empowerment. As I watched Rahima and Ghazal tackle the situation in a thoroughly professional manner, I realized that they will be my future colleagues.They are the brilliant future physicians that I expected this medical school to foster, and we have united to speak up for the sake of a stronger, more ethically sensitive community.

## Conclusion

Dealing with provocative comments, racism and other forms of bigotry in a medical school is nuanced and risky. Having a framework for how to approach these situations can be helpful, given the inherent uncertainty in any crisis. The following are four key lessons that we learned through this process:

1. Students’ experiences of provocative comments and their attitudes toward leadership’s response can be quite different than what leaders might imagine. Recognizing this possibility can help leaders pause before taking action.
2. Leaders must be open to what students have to say, even when it hurts. Responding to student frustration with curiosity and a commitment to engage even in the most difficult conversations can be more effective than merely citing policy and procedure.
3. There is tension between a learning institution’s mission of helping students develop, particularly when they engage in unprofessional behaviors, and the sense of urgency for action in response to provocative. A collaborative approach integrating students in the process can help students and leaders tolerate intense emotions and uncertainty.
4. Collaborating with students to gain their perspectives on difficult situations can help leaders to deliver unpleasant messages in a more direct, yet tolerable, manner.

Developing a trusting and open relationship between administration and student leaders is crucial to managing a crisis. While our particular situation was never fully resolved, jointly building a collaborative relationship as the crisis unfolded resulted in increased trust and respect within our educational community.

## Take Home Messages

• Students and medical school leaders experience provocative comments differently.
• Medical school leaders must respond to student frustration with curiosity and openness.
• Collaborating with students, and actively integrating their perspectives in resolving hate speech issues, can decrease tension around incidents and provide a roadmap for leaders to communicate with all students in direct and helpful ways.

## Notes On Contributors

Kimberly R. Myers, M.A., Ph.D., is a Professor of Humanities and Medicine at the Penn State College of Medicine.

Rahima Khatun is a third-year medical student at the Penn State College of Medicine.

Ghazal Staity is a third-year medical student at the Penn State College of Medicine.

Shreela Natarajan is a third-year medical student at the Penn State College of Medicine.

Daniel R. George, Ph.D., is an Associate Professor of Humanities at the Penn State College of Medicine.

George F. Blackall, Psy.D., MBA, is the Director of the Office for a Respectful Learning Environment and Professor of Pediatrics and Humanities at the Penn State College of Medicine.
