# Supplementary material for: A Collaborative Response to Provocative Comments in a Medical School
Source: MedEdPublish (2016). 2019 Nov 6;8:199. [Version 1] doi: 10.15694/mep.2019.000199.1 (PMC10712520; doi:10.15694/mep.2019.000199.1)
Supplement: Peer review history

# Reviewer report: Reviewer response for version 1

**DOI:** 10.21956/mep.19896.r30113

**Published:** 2019-11-15

**Authors:** Sateesh Babu Arja

**Affiliations:** 1 Avalon University School of Medicine

This review has been migrated. The reviewer awarded 4 stars out of 5 It is very interesting paper . It is very unfortunate that bias exists in medical schools based on age, sex, religion, and race/ethnicity even though this medical profession must be the most tolerant one. The maintenance of professional behaviors in academic environment and safe learning environment are very essential. It is nice to see Penn state medical school has the Office for a Respectful Learning Environment (ORLE). It is surprising for me to see that Academic Progress Committee also handles the professional issues along with academic issues based on my background. In the context of where I am working, medical schools have two different committees for Academic progression and Disciplinary/ professional issues. The authors have explained very clearly how this situation was handled and the educational leaders have handled this situation very appropriately by holding meetings with stakeholders. The stakeholders were informed adequately. This manuscript is important for all academic administrators and educational leaders.

Reviewer Expertise:

NA

No decision status is available

# Reviewer report: Reviewer response for version 1

**DOI:** 10.21956/mep.19896.r30114

**Published:** 2019-11-06

**Authors:** Judy McKimm

**Affiliations:** 1 Swansea University

This review has been migrated. The reviewer awarded 4 stars out of 5 I read this with interest as sadly this is not an uncommon occurrence in medical schools, with hate directed towards both students and faculty on various grounds. This has been discussed from a range of perspectives and I really like how different groups' and individuals' voices have been heard. The take home points are also very useful, various stakeholders do see events very differently and I like the openness of the article, e.g. in opening up class discussions which can sometimes lead to unintended and unforeseen consequences. I think this would be very useful for schools encountering such incidents.

Reviewer Expertise:

NA

No decision status is available
